# 盐酸米托蒽醌脂质体联合阿糖胞苷治疗RUNX1∷MTG16融合基因阳性儿童急性髓系白血病1例及文献复习

**DOI:** 10.3760/cma.j.cn121090-20240805-00289

**Published:** 2024-12

**Authors:** 硕 林, 本泉 戚, 立鹏 刘, 继刚 肖, 文钰 杨, 晓凡 竺, 晓娟 陈

**Affiliations:** 1 中国医学科学院血液病医院（中国医学科学院血液学研究所）、血液与健康全国重点实验室、国家血液系统疾病临床医学研究中心、细胞生态海河实验室，天津 300020 Institute of Hematology & Blood Diseases Hospital, Chinese Academy of Medical Sciences, State Key Laboratory of Experimental Hematology, National Clinical Research Center for Blood Diseases, Haihe Laboratory of Cell Ecosystem, Tianjin 300020, China; 2 天津医学健康研究院，天津 301600 Tianjin Institutes of Health Science, Tianjin 301600, China

## Abstract

回顾性分析中国医学科学院血液病医院2023年10月收治的1例急性髓系白血病（AML）伴RUNX1∷MTG16患儿，男性，13岁，因“乏力20 d”就诊，骨髓细胞形态学可见17.0％原始细胞，染色体核型46,XY,t（16;21）（q24;q22），RUNX1∷MTG16融合基因阳性，合并FLT3-ITD突变，诊断为AML（伴RUNX1∷MTG16）。经盐酸米托蒽醌脂质体联合阿糖胞苷（MA）方案诱导化疗达到完全缓解，序贯MA方案1个疗程后RUNX1∷MTG16融合基因及FLT3-ITD突变均转阴。但继续巩固强化治疗中融合基因及突变均转为阳性，行同胞全相合脐血造血干细胞移植存活。

## Abstract (en)

This case report presents a patient with pediatric acute myeloid leukemia（AML）with RUNX1∷MTG16, admitted to the Blood Disease Hospital of the Chinese Academy of Medical Sciences in October 2023. He was 13 years old, with a chief complaint of fatigue for 20 days. Bone marrow smear revealed 17.0％ blasts, the karyotype was 46,XY,t（16;21）（q24;q22）, molecular biology demonstrated RUNX1∷MTG16 fusion gene, combined with FLT3-ITD mutation. The child was diagnosed with AML（with RUNX1∷MTG16）. Complete remission was achieved after chemotherapy induction. The induction therapy regimen was mitoxantrone hydrochloride liposomes combined with cytarabine（MA）. The RUNX1∷MTG16 and FLT3-ITD were negative after another MA treatment course. However, the RUNX1∷MTG16 and FLT3-ITD were turning positive during the following intensive treatment, and he then successfully underwent matched sibling donor umbilical cord blood transplantation.

急性白血病是儿童最常见的血液系统恶性肿瘤，其中急性髓系白血病（AML）约占儿童白血病的25％[1]，核心结合因子相关AML（CBF-AML）是儿童AML中常见细胞遗传学亚型，占25％～30％[2]。RUNX1∷MTG16融合基因是由t（16;21）（q24;q22）染色体核型异常形成，发生率较低，罕见于儿童AML，研究认为具有该细胞遗传学特点的AML是CBF-AML中少见的亚组，此类患者大多具有FAB-M_1_/M_2_形态表型[3]。目前WHO尚未对此类AML进行特殊分类，尚无标准的治疗方案。我们回顾性分析1例我院收治的伴RUNX1∷MTG16融合基因AML患儿的临床特征及治疗并进行文献复习，加深对儿童AML伴RUNX1∷MTG16这一少见类型疾病诊疗的认识。

## 病例资料

患儿，男，13岁，主因“乏力20 d”收治于中国医学科学院血液病医院儿童血液与肿瘤诊疗中心。患儿入院前20 d受凉后出现乏力、头晕，伴有憋气，活动后加重，后上述症状逐渐加重，出现齿龈出血。就诊当地医院查血常规示WBC 6.34×10^9^/L，HGB 39 g/L，PLT 18×10^9^/L，予成分血输血治疗后入我院进一步诊治。患儿系其母第1胎第1产，足月自然出生，生后肛门闭锁，3岁时行肛门扩大手术，否认父母近亲婚配及家族遗传病史。

入院体格检查：体温36.4 °C，脉搏94次/min，呼吸25次/min，血压115/60 mmHg（1 mmHg＝0.133 kPa）。发育正常，营养中等，神志清醒，中度贫血貌，结膜苍白，巩膜无黄染，齿龈无红肿。周身皮肤无皮疹、黄染，无瘀点、瘀斑，浅表淋巴结未触及肿大，胸骨无压痛，腹软、无压痛及反跳痛，肝脾肋缘下未触及，肛门及外生殖器无外观改变，双下肢无水肿，病理征阴性。

辅助检查：血常规示WBC 3.89×10^9^/L，中性粒细胞计数1.19×10^9^/L，RBC 2.28×10^12^/L，HGB 73 g/L，PLT 8×10^9^/L。生化检查示铁蛋白494.80 ng/ml。骨髓涂片示原始细胞比例17.0％，外周血涂片示原始细胞比例6％。流式细胞术检查示异常细胞群占有核细胞的7.11％；髓系原始细胞比例增高，表型异常；粒系核左移，以中幼粒细胞为主，CD13/CD16、CD13/CD11b分化抗原表达异常；强表达CD117，表达CD34、HLA-DR、CD33，弱表达CD13、CD123、CD38、MPO。分子生物学检查融合基因筛查示RUNX1∷MTG16阳性，FLT3-ITD等位基因比率0.04，U2AF1基因p.S34Y突变。

患儿骨髓细胞形态学示原始幼稚细胞17％，融合基因RUNX1∷MTG16阳性，根据WHO 2022 AML标准，诊断为具有重现性基因异常的AML（伴RUNX1∷MTG16，FLT3-ITD）。

患儿应用盐酸米托蒽醌脂质体联合阿糖胞苷（MA）方案，诱导治疗后骨髓细胞形态学达完全缓解（CR），融合基因RUNX1∷MTG16阳性，FLT3-ITD等位基因比率：0.00，U2AF1阴性，流式细胞术检测MRD未见异常髓系表型，血常规示PLT>100×10^9^/L，中性粒细胞<1×10^9^/L，评估治疗反应为CR伴不完全血细胞恢复（CRi）；序贯MA方案巩固化疗1个疗程后骨髓仍为CR，流式细胞术检测MRD未见异常髓系表型，RUNX1∷MTG16、FLT3-ITD及U2AF1均为阴性。MA方案化疗过程中有Ⅰ级恶心、呕吐等不良反应，未发生严重感染，化疗耐受性良好。考虑患儿两个疗程MA方案化疗后骨髓增生减低，中性粒细胞持续减低，后续应用HAG方案巩固化疗，监测RUNX1∷MTG16、FLT3-ITD转为阳性，骨髓免疫分型MRD示原始细胞0.13％，考虑存在复发趋势。患儿与其同胞妹妹HLA配型12位点全相合，最终成功行同胞全相合脐血造血干细胞移植，移植后第21天粒细胞植入，第26天血小板植入，复查RUNX1∷MTG16阴性，FLT3-ITD等位基因比率：0.00，U2AF1阴性，流式细胞术检测MRD未见异常髓系表型，目前患儿为移植后2个月余，血常规正常，无病存活。

## 讨论及文献复习

随着诊断及治疗技术的不断发展，儿童AML的预后有所改善，目前生存率高达70％以上[1],[4]。CBF-AML是儿童AML中常见细胞遗传学亚型，占25％～30％[2]，一般预后良好[5]，主要包括RUNX1∷RUNX1T1和CBFβ∷MYH11两种特征性重现性融合基因异常[6]。RUNX1∷MTG16融合基因是由t（16;21）（q24;q22）染色体核型异常形成，类似于t（8;21）（q22;q22）形成的RUNX1∷RUNX1T1融合基因，两者RUNX1基因的断裂点均位于第5、6外显子之间[7]，除了共享RUNX1基因，MTG16与RUNX1T1作为同一蛋白家族成员，同源性高达70％[8]。RUNX1∷MTG16与RUNX1∷RUNX1T1具有相似的生物活性、基因表达谱[9]、形态学和表型特征[10]。I-BFM协作组[3]研究证实RUNX1∷MTG16在儿童AML发病率较低，4年无事件生存（EFS）率为77％，累计复发率为0，提示此类患儿预后良好，认为RUNX1∷MTG16属于CBF-AML亚组，可从标准风险分层的治疗中受益。

本例患儿RUNX1∷MTG16融合基因阳性，同时合并FLT3-ITD突变。研究认为，携带FLT3-ITD突变的CBF-AML患者总生存（OS）时间及无复发生存（RFS）时间短，与较差的预后和高复发率相关[11]–[12]，不建议归入低危组类。结合本例患儿治疗反应，考虑伴RUNX1∷MTG16儿童AML合并有FLT3-ITD突变者更易产生化疗耐药导致不良预后。同时患儿初诊时检测到U2AF1突变，该突变常见于髓系恶性肿瘤，促进白血病转化，预示AML不良预后[13]–[14]。值得注意的是，患儿有肛门闭锁病史，肛门闭锁作为胚胎发育早期出现异常的结果，人群发病率1/1 500～1/5 000[15]，发病因素可能与遗传有关[16]。先天性骨髓衰竭表现为进行性骨髓造血衰竭，通常伴有躯体畸形及肿瘤易感，其中躯体畸形表现包括肛门闭锁[17]–[18]。因此，伴有躯体畸形的AML需警惕先天性骨髓衰竭性疾病而AML易感，此患儿行靶向二代测序未发现与先天性骨髓衰竭确切相关的基因突变，患者既往无血细胞减少病史，无先天性骨髓衰竭疾病及肿瘤家族史，目前暂不考虑患者存在先天性骨髓衰竭疾病，未行全基因组检测，但在疾病进程中需密切随访患者病情变化，必要时行全基因组检测进一步排除先天性骨髓衰竭性疾病。

儿童AML诱导治疗常采用以阿糖胞苷和蒽环类药物为基础的联合治疗方案。米托蒽醌作为AML治疗经典的蒽环类药物，可引起DNA结构的交联和断裂、干扰RNA，同时也是拓扑异构酶Ⅱ的有效抑制剂[19]，可抑制肿瘤细胞增殖、诱导肿瘤细胞凋亡。EORTC和LAME协作组等研究报道，与柔红霉素相比，米托蒽醌可提高患者CR率和延长无病生存（DFS）期，缓解程度更深，且诱导治疗期间的死亡率无显著差异[20]–[21]。MRC AML12协作组证实米托蒽醌相比于柔红霉素可改善AML患儿DFS[22]，但心脏毒性仍限制其临床应用[23]。Lipo-MIT通过对米托蒽醌进行脂质体包裹改良，是普通米托蒽醌的改良和升级的新剂型[24]，Lipo-MIT没有改变米托蒽醌固有的药理作用机制，但在药物到达肿瘤组织前保持脂质体完整性，减少心脏毒性和骨髓抑制等不良反应的发生[25]，具有良好的抗肿瘤效果和安全性，有助于改善远期生存[25]–[27]，国内单中心研究表明含Lipo-MIT方案治疗高危儿童AML疗效与去甲氧柔红霉素相当，但临床安全性更佳[26]。Tierens等[28]研究证实，应用Lipo-MIT治疗可以降低患儿累积复发率（CIR），提高EFS率，改善患儿预后，相较于柔红霉素脂质体具有更好的抗白血病效果。

本例患儿应用MA方案可达分子生物学CR，疗效肯定，且不良反应小，可耐受。MA方案诱导化疗及巩固化疗1个疗程后骨髓象CR，细胞遗传学异常均转阴，流式细胞术MRD阴性，结合血常规，评估为CRi，表明患儿应用MA方案治疗获益显著，治疗期间患儿曾短暂出现与Lipo-MIT用药相关的头痛及发热，可自行缓解，并发现皮肤蓝染，以颜面部及四肢远端的皮肤为著，无疼痛、皮肤溃烂等，证实Lipo-MIT药物安全性良好。Chen等团队研究表明治疗后CRi患者相较于CR患者具有较差预后[29]–[30]，此患儿在巩固治疗过程中融合基因及基因突变由阴性转为阳性，最终行同胞全相合脐血造血干细胞移植治疗，病情得到控制。

目前国内外对AML伴RUNX1∷MTG16患者尚无规范治疗方案，既往研究认为此类患者可从标准风险分层的治疗中受益，但尚无合并FLT3-ITD突变患者的报道及预后研究。MA方案诱导化疗耐受性良好，无明显不良反应，诱导缓解率高，可作为伴RUNX1∷MTG16儿童AML的推荐诱导化疗方案，对合并有FLT3-ITD突变者更易产生化疗耐药，造血干细胞移植可改善预后。
